# Case report: Extrapontine myelinolysis combined with flupentixol- and melitracen-induced dysphagia

**DOI:** 10.3389/fphar.2023.1266296

**Published:** 2023-10-18

**Authors:** Zhihong Zhao, Lu Han, Zilong Zhu

**Affiliations:** ^1^ Department of Neurology, Tianjin Huanhu Hospital, Tianjin, China; ^2^ Department of Electroencephalogram, Tianjin Huanhu Hospital, Tianjin, China

**Keywords:** extrapontine myelinolysis, flupentixol and melitracen, hyponatremia, dysphagia, sodium

## Abstract

Extrapontine myelinolysis (EPM) is a rare symmetrical demyelinating disease of the central nervous system, which is often accompanied with central pontine myelinolysis (CPM) or can appear alone. A combination of flupentixol and melitracen is used as an antianxiety–antidepressant drug which may induce hyponatremia. Herein, we report a 46-year-old woman with depression who was treated with flupentixol and melitracen 0.5/10 mg once daily for 6 months. Later, the dosage increased to 0.5/10 mg twice daily. At the same time, she had complains of intermittent dizziness and fatigue. The laboratory test revealed hyponatremia (121 mmol/L). Dizziness was improved after sodium supplementation, with an increase in blood sodium to 133 mmol/L. Twenty days later, she had difficulty opening the mouth and swallowing, needing a gastric tube due to severe dysphagia. Head magnetic resonance imaging (MRI) showed a symmetric abnormal signal of caudate nucleus and lenticular nuclei. The symptoms were not relieved after active treatment, such as rehydration. However, her symptoms improved significantly after discontinuation of flupentixol and melitracen and switching to promethazine. Follow-up head MRI after 4 months revealed no abnormal signals. The patient who developed EPM had dysphagia, despite appropriate correction of hyponatremia. Flupentixol and melitracen can cause hyponatremia and dysphagia. This case highlights an unexpected association between EPM and flupentixol- and melitracen-induced dysphagia.

## Introduction

Extrapontine myelinolysis (EPM) is a rare, symmetrical, non-inflammatory disorder characterized by demyelination that is often concomitant with central pontine myelinolysis (CPM) or can appear alone (Singh et al., 2014). A combination of flupentixol and melitracen is widely used clinically as an antianxiety–antidepressant drug (Chen et al., 2021). Flupentixol increases the incidence of extrapyramidal side effects, such as akathisia, limb tremor, and involuntary movements, which can develop with long-term use. However, there have been few reports of dysphagia (Zahran et al., 2023). Herein, we present an interesting case that dysphagia is caused by both EMP and flupentixol and melitracen.

## Case report

**FIGURE 1:**
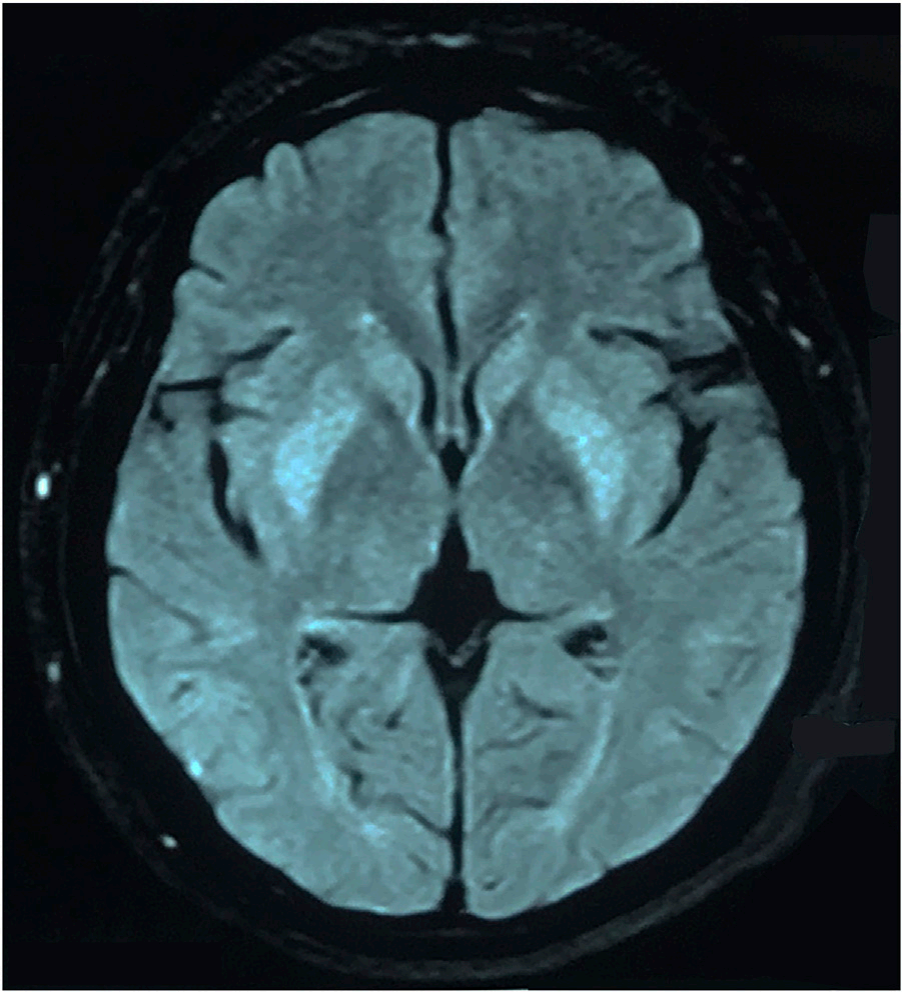
Head MRI revealed symmetric FLAIR hyperintensities of the bilateral basal ganglia.

**FIGURE 2:**
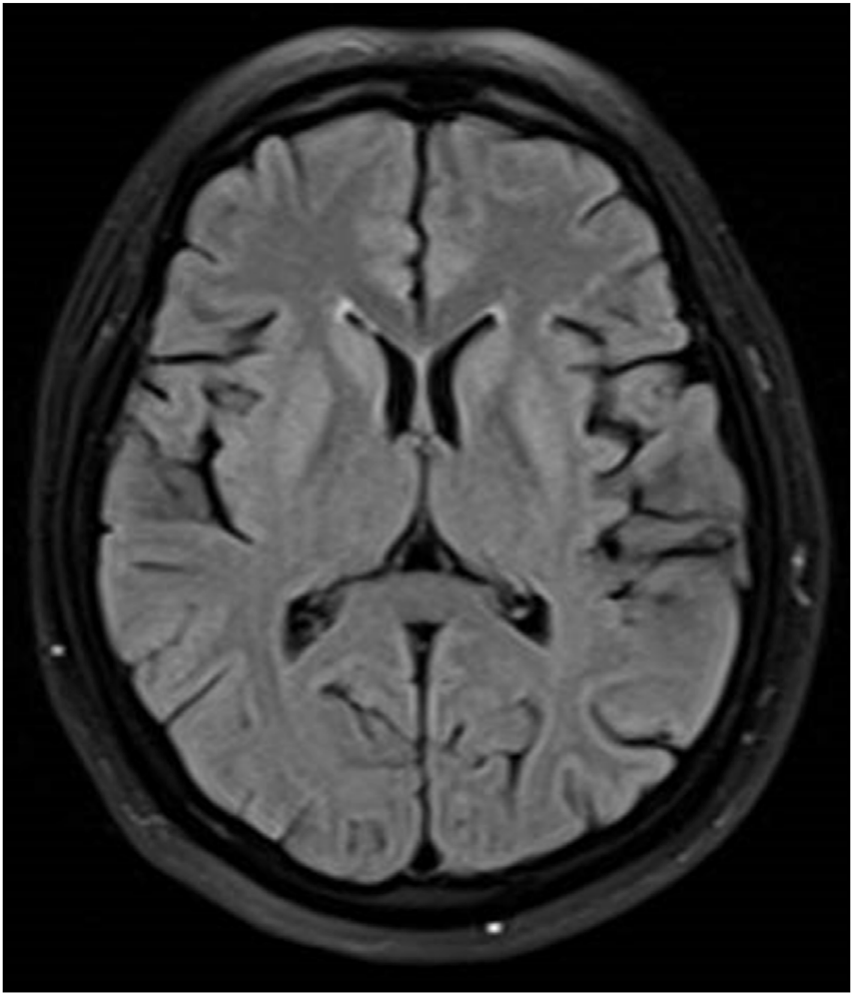
Resolution of bilateral FLAIR hyperintensities before discharge.

**FIGURE 3:**
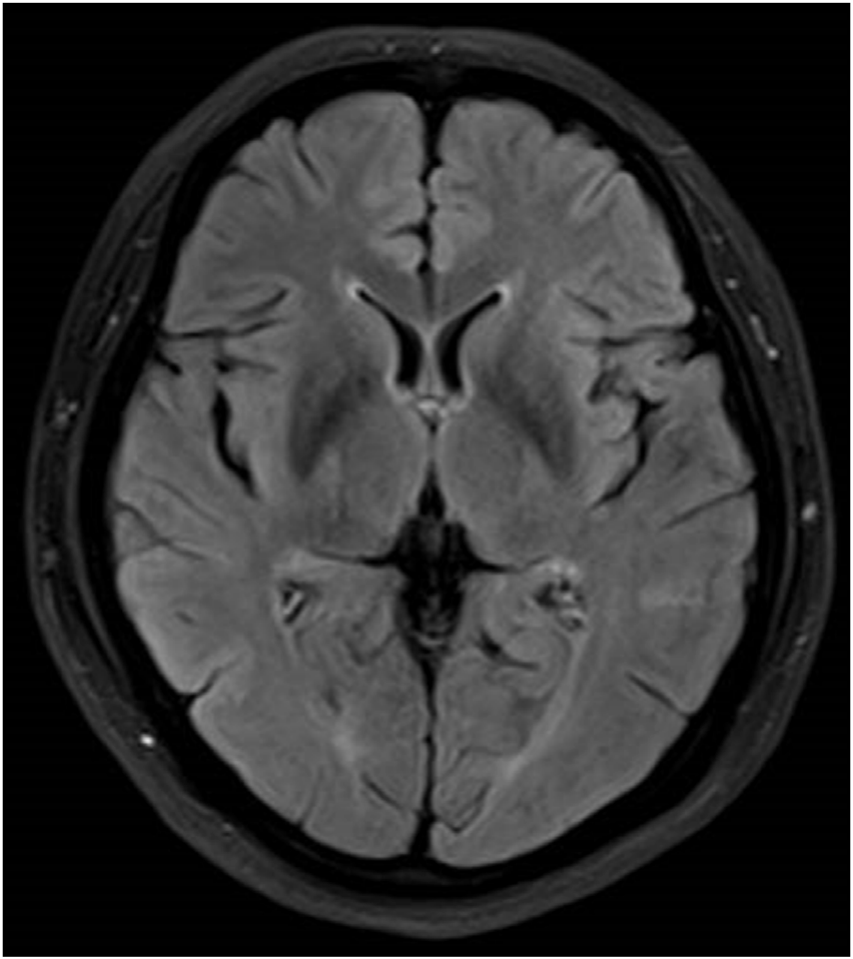
No abnormal signals in the bilateral basal ganglia after 4 months.

The patient was a 46-year-old woman who was admitted to our department with the chief complaint of “difficulty in opening the mouth and swallowing for 16 days.” The patient had a history of depression for 6 months and was treated with flupentixol and melitracen 0.5 mg/10 mg once daily, which was later increased to 0.5 mg/10 mg twice daily for a month until diagnosed with dysphagia. No other drugs were taken by the patient, and family history was denied. Toxic exposure was denied. She also had complains of intermittent mild dizziness and fatigue for the last 6 months. The laboratory test revealed hyponatremia. The patient’s serum sodium level was not less than 130 mmol/L. Her dizziness improved after salt intake. The doctor did not predict that it was an adverse reaction to flupentixol and melitracen. Twenty days before the onset of dysphagia, the patient’s serum sodium level due to moderate fatigue was found to be 121 mmol/L. An increase in the serum sodium level to 133 mmol/L was observed after sodium supplementation for 11 days. The patient received normal saline intravenously in the first 24 h, and the serum sodium level rose to 126 mmol/L. She continued to receive saline intravenously for 2 days, and the serum sodium level rose to 131 mmol/L. The serum sodium level further rose to 133 mmol/L after taking salt orally. Sixteen days before admitting to our hospital, the patient presented with difficulty in chewing and opening the mouth, dysphagia with excessive salivation, and slurred speech. These symptoms progressed to inability to swallow. There was no limb weakness. The patient sought treatment at the local hospital, where she received nasogastric feeding because of dysphagia. Head magnetic resonance imaging (MRI) showed symmetrical hyperintensities on the fluid-attenuated inversion recovery (FLAIR) images over bilateral caudate nucleus and lenticular nucleus (Figure 1). Laboratory tests revealed that the serum sodium level was 124 mmol/L, which increased to 139 mmol/L after sodium supplementation for 2 days. No abnormalities of the pituitary hormones, tumor biomarkers, glucose, or thyroid function were observed. No abnormality was found in hepatic and renal function. The patient was diagnosed with EPM and received methylprednisolone 500 mg once daily for 3 days, followed by 250 mg once daily for 3 days and 120 mg once daily for 2 days, in addition to neurotrophic drugs. However, the patient’s symptoms did not improve, and she was transferred to our hospital. Neurological examination revealed severe dysarthria, weak tongue extension with inability to extend it outside the mouth, weak soft palate, and bilateral loss of pharyngeal reflexes. The serum sodium level was 139 mmol/L. No abnormalities of serum ammonia, copper, and ceruloplasmin were observed. EPM was considered based on the patient’s medical history and imaging results. Treatment was provided to improve the cerebral metabolism and provide nutritional support and rehydration, while flupentixol and melitracen treatment was continued at 0.5 mg/10 mg once daily. Hyperbaric oxygen therapy was initiated on the third day after admission, but the patient’s symptoms did not improve. Flupentixol and melitracen was tapered on the sixth day and discontinued after 2 days, and the patient was switched to intramuscular injection of promethazine (50 mg once daily). Subsequently, the patient’s dysphagia and dysarthria improved significantly. On the ninth day after admission, the patient could eat but occasionally choked when drinking water. Head MRI re-examination on day 13 demonstrated significant resolution of the bilateral basal ganglia on the FLAIR images (Figure 2). The patient no longer choked when drinking water, and the nasogastric tube was removed. Follow-up head MRI after 4 months revealed no abnormal signals (Figure 3). Blood electrolyte examination showed no abnormality, and the serum sodium level was 142 mmol/L. The patient spoke fluently, and she extended her tongue, opened her mouth, and swallowed normally. All patient details were de-identified for this case report.

## Discussion

Herein, we report a patient presenting with dysphagia who was found to have imaging features of EPM as well as dysphagia caused by flupentixol and melitracen.

CPM was first described as non-inflammatory demyelination centered within the basis pontis. CPM with/without EPM is a well-recognized complication of rapid correction of hyponatremia. EPM rarely appears alone. The most common site of EPM is the basal ganglia, although the cerebral cortex, thalamus, and cerebellum may also be involved (Martin, 2004). The common causes include hyponatremia with rapid correction, followed by chronic alcoholism, hepatic and renal failure, and cancer (Aoki et al., 2016). The underlying pathophysiological mechanisms remain elusive, which may be related to the imbalance of osmotic pressure in the brain (Lambeck et al., 2019). The clinical manifestations of EPM depend on the site of involvement as well as the extent of the lesion. Symptoms such as dystonia, ataxia, Parkinsonism, or mental and behavioral abnormalities may occur (Ho et al., 2019). In this study, the patient only presented with dysphagia, which has rarely been reported in previous cases.

While correcting hyponatremia, the rate of sodium supplementation should be strictly controlled. According to the 2014 European clinical practice guidelines on the management of hyponatremia, the increase in serum sodium concentration while treating moderate or severe hyponatremia is restricted to no more than 10 mmol/L during the first 24 h, followed by an increase of less than 8 mmol/L every 24 h thereafter until the serum sodium reaches 130 mmol/L (Spasovski et al., 2014). Tolerance to hyponatremia differs among patients. There have been clinical reports of EPM, despite sodium supplementation rates being within the required range (Micieli et al., 2020). Excessive or excessively rapid sodium supplementation should be paid attention to for patients with chronic hyponatremia. In the present case, there was no rapid correction of the patient’s sodium, but EPM still occurred, suggesting that chronic hyponatremia may result in varying degrees of reduced neuronal tolerance to changes in osmotic pressure. More caution is required while correcting chronic hyponatremia.

Flupentixol and melitracen is a combination including flupentixol and melitracen. Long-term use can result in extrapyramidal symptoms, in addition to drowsiness, loss of appetite, and dizziness. Flupentixolisa butylbenzene, an antipsychotic, exerts very different effects at large and small doses. Small doses of flupentixol promote the release of dopamine, whereas large doses inhibit dopamine activity. Flupentixol strongly blocks the D2 dopamine receptors, thus increasing the incidence of extrapyramidal side effects (Joubert et al., 2021). Melitracen inhibits the presynaptic membrane reuptake of serotonin and norepinephrine and increases the levels of monoamine transmitters in the synaptic cleft. Hyponatremia can be caused by selective serotonin reuptake inhibitors, which may stimulate an increase in the release of the antidiuretic hormone (Levine et al., 2017; Wang et al., 2018). The syndrome of inappropriate antidiuresis (SIAD) is the commonest cause of hyponatremia. SIAD occurs in a wide variety of diseases, such as malignant tumor, drugs, brain surgery, and rheumatic diseases. The mechanism of SIAD induced by antidepressants may be mediated by the action of 5-selective serotonin. Only one domestic case of hyponatremia and dysphagia caused by flupentixol and melitracen has been reported, and the symptoms improved significantly after discontinuation of the drug (Hou and Zhou, 2012). Studies abroad have also reported pharyngeal dystonia and consequent dysphagia after flupentixol treatment (Agarwal and Ichaporia, 2010; Tang and Hsieh, 2010). In this case, the patient received flupentixol and melitracen for 6 months, and the dosage was increased later. The patient developed severe dysarthria and dysphagia, and her symptoms improved significantly after discontinuation of the drug and switching to promethazine, which inhibited dopamine receptors that were blocked.

The images of EPM and toxic encephalopathy appear similar, and sometimes, it is difficult to distinguish. Head MRI showed bilateral FLAIR symmetric hyperintense lesions in brain stem, cerebellum, basal ganglia, and subcortical white matter. We could see the classical appearance of trident- or batwing-shaped central pontine abnormality in CPM. Concomitant or isolated involvement of extrapontine sites maybe seen, including basal ganglia, midbrain, thalami, cerebellum, and cerebral white matter. However, this is very significant that diffuse, symmetric, and bilateral hyperintensities may be perceived in the subcortical or periventricular white matter in toxicosis. CPM and EPM are caused by various toxic exposure events including medications, illicit drug use, and environmental exposures. In addition, it also needs to be differentiated from other metabolic diseases and endocrine diseases. This patient had a history of recurrent hyponatremia and electrolyte correction. We have carried out laboratory tests for thyroid, pituitary hormones, blood ammonia, and ceruloplasmin to rule out other diseases. Therefore, it is important to highlight that the diagnosis is not straightforward, and adequate correlation with clinical history and laboratory data is essential for accurate assessment.

Hyponatremia may have occurred in this patient as she received flupentixol and melitracen for a long period of time with a recent increase in the dosage, and she consumed less food daily. However, EPM developed despite strict control of the rate of sodium supplementation. The patient’s dysphagia appeared to improve significantly after discontinuation of flupentixol and melitracen and switching to promethazine. It is noteworthy that the patient’s symptoms improved, along with disappearance of the abnormal signals in the bilateral basal ganglia on MRI re-examination. We speculated that dysphagia was caused by both the extrapyramidal effects of previous EPM and pharyngeal dystonia caused by flupentixol and melitracen, with the latter being the principal cause in the case.

Hyponatremia and dysphagia may occur after the long-term use of flupentixol and melitracen. Regular neurological examination is needed to detect adverse drug reactions in time. Patients with a slow correction of hyponatremia may also develop central pontine with/without extrapontine myelinolysis.

## Data Availability

The original contributions presented in the study are included in the article/Supplementary Material; further inquiries can be directed to the corresponding author.
